# Anti-inflammatory and Oto-Protective Effect of the Small Heat Shock Protein Alpha B-Crystallin (HspB5) in Experimental Pneumococcal Meningitis

**DOI:** 10.3389/fneur.2019.00570

**Published:** 2019-06-10

**Authors:** Silvia T. Erni, Gabriella Fernandes, Michelle Buri, Michael Perny, Rolf Jan Rutten, Johannes M. van Noort, Pascal Senn, Denis Grandgirard, Marta Roccio, Stephen L. Leib

**Affiliations:** ^1^Neuroinfection Laboratory, Institute for Infectious Diseases, University of Bern, Bern, Switzerland; ^2^Cluster for Regenerative Neuroscience, DBMR, University of Bern, Bern, Switzerland; ^3^Laboratory of Inner Ear Research, DBMR, University of Bern, Bern, Switzerland; ^4^Graduate School for Cellular and Biomedical Sciences, University of Bern, Bern, Switzerland; ^5^Audion Therapeutics, Amsterdam, Netherlands; ^6^Delta Crystallon BV, Leiden, Netherlands; ^7^Service d'oto-rhino-laryngologie (ORL) et de chirurgie cervico-faciale, Département des Neurosciences Cliniques, Hôpitaux Universitaires de Genève, Geneva, Switzerland; ^8^Department of Otorhinolaryngology, Head & Neck Surgery, Inselspital, Bern, Switzerland

**Keywords:** sensorineural hearing loss, *S.pneumoniae*, HspB5, inflammation, oto-protection, hair cells, neutrophil infiltration

## Abstract

Sensorineural hearing loss is the most common long-term deficit after pneumococcal meningitis (PM), occurring in up to 30% of surviving patients. The infection and the following overshooting inflammatory host response damage the vulnerable sensory cells of the inner ear, resulting in loss of hair cells and spiral ganglion neurons, ultimately leading to elevated hearing thresholds. Here, we tested the oto-protective properties of the small heat shock protein alpha B-crystallin (HspB5) with previously reported anti-inflammatory, anti-apoptotic and neuroprotective functions, in an experimental model of PM-induced hearing loss. We analyzed the effect of local and systemic delivery of HspB5 in an infant rat model of PM, as well as *ex vivo*, using whole mount cultures. Cytokine secretion profile, hearing thresholds and inner ear damage were assessed at predefined stages of the disease up to 1 month after infection. PM was accompanied by elevated pro-inflammatory cytokine concentrations in the cerebrospinal fluid (CSF), leukocyte and neutrophil infiltration in the perilymphatic spaces of the cochlea with neutrophils extracellular trap formation during the acute phase of the disease. Elevated hearing thresholds were measured after recovery from meningitis. Intracisternal but not intraperitoneal administration of HspB5 significantly reduced the levels of TNF-α, IL-6 IFN-γ and IL-10 in the acute phase of the disease. This resulted in a greater outer hair cell survival, as well as improved hearing thresholds at later stages. These results suggest that high local concentrations of HspB5 are needed to prevent inner ear damage in acute PM. HspB5 represents a promising therapeutic option to improve the auditory outcome and counteract hearing loss after PM.

## Introduction

Worldwide around 460 million people suffer from disabling hearing loss (world health organization fact sheet 2019). This is caused by inherited genetic defects or acquired damage by ototoxic drugs, exposure to excessive noise, aging or certain infectious diseases such as meningitis. *Streptococcus pneumoniae* is the most common and simultaneously the most severe pathogen causing bacterial meningitis. Pneumococcal meningitis (PM) is associated with high mortality and risk for neurological sequelae, with sensorineural hearing loss representing the most frequent long term deficit (1) occurring in up to 30% of surviving patients (2).

Bacterial meningitis evokes a robust inflammatory response marked by recruitment of leukocytes, mostly polymorphonuclear neutrophils, to the cerebrospinal fluid (CSF) spaces, including the inner ear. For many years the cochlea was thought to be immune-privileged, but more recent studies have shown that resident macrophages are present in the lateral wall, in the spiral limbus and in the basal side of basilar membrane and are activated after insult (3–7).

Upon infection, pro-inflammatory cytokines such as TNFα, IL-1β, IL-6, IL-8 are secreted in the CSF (8, 9) and pneumococci and leukocytes infiltrate the perilymphatic space of the inner ear via the cochlear aqueduct (1, 6, 10). These processes, together with the release of the bacterial exotoxin pneumolysin, cause an accentuated inflammation of the inner ear, especially in the basal portion of the cochlea (11, 12). This results in histopathological changes of the sensory epithelium and associated structures, which are crucial for the hearing process. Spiral ganglion neuron loss and outer hair cell loss strongly correlate to the level of pneumococcal infection, with the most severe damage occurring in the basal part of the cochlea (8). Inner hair cells are not significantly lost but the number of presynaptic ribbons was shown to be decreased (8). Increased hearing thresholds are already observed during the acute phase of experimental pneumococcal meningitis and in most cases persist until later stages (8, 12–14).

Current treatment options for sensorineural hearing loss consist of hearing aids and cochlear implants, both relying for their effectiveness on remaining intact hair cells and/or spiral ganglion neurons (15). Novel therapeutics are highly needed to protect these cells in the first place or to induce a regenerative response.

Heat shock proteins have already been proposed as therapeutic targets in various diseases (16). Activation of heat shock proteins is the most ubiquitous and highly conserved stress response in biology (17, 18).

Specific to the inner ear, May et al. observed a protective effect of Hsp70 against aminoglycoside-induced hair cell death *in vitro* and *in vivo* (19). Additionally, expression of Hsp70 and induction of Hsp32 offered protection against cisplatin-induced hair cell death in utricle cultures (20).

Over the last decade, evidence has accumulated for the broad neuroprotective and anti-inflammatory activity of HspB5, also known as alpha B-crystallin or CRYAB (21, 22). Several studies demonstrated HspB5-mediated cell protection under various stress conditions (23) such as experimental autoimmune encephalomyelitis (24), stroke (25) and Alexander disease (26).

HspB5 is a member of the small heat shock family that is constitutively expressed in many tissues and especially abundant in eye, lens, heart, muscle and oligodendrocytes of the CNS (21, 23). In pathological conditions such as multiple sclerosis and brain ischemia it is also expressed in other glial cells (21, 27). HspB5 is stress-inducible (22) and has anti-apoptotic (28, 29), anti-inflammatory (21) and neuroprotective (30) functions.

Different mechanisms of action have been reported for HspB5 in the literature. The anti-inflammatory effect of HspB5 has been shown to be associated with activation of an immune-regulatory macrophage response via Toll-like receptors (TLR1/2) and the co-receptor CD14 (31, 32). Its chaperone activity, by binding to misfolded proteins also contributes to its anti-inflammatory activity (24, 26). Its anti-apoptotic effects have been instead attributed to direct interaction with pro-apoptotic molecules, such as Caspase-3, or by interfering with cytochrome C release from the mitochondria (33).

In a murine model of stroke, Cryab^−/−^ mice had increased lesion size and diminished neurologic function compared with wild-type mice. Even 12 h after experimental stroke, systemic administration of HspB5 reduced both stroke volume and inflammatory cytokines associated with stroke pathology (25).

Furthermore, in a model of experimental autoimmune encephalomyelitis Cryab^−/−^ mice showed worse scores in the acute and in the progressive phases, with higher Th1 and Th17 cytokine secretion and more intense CNS inflammation compared with their wild-type counterparts. Moreover, Cryab^−^/^−^ astrocytes showed increased expression of cell death markers, such as cleaved caspase-3 and DNA breaks, detected by TUNEL staining, indicating an anti-apoptotic function of HspB5. Administration of recombinant HspB5 ameliorated experimental autoimmune encephalomyelitis (21).

A phase IIa randomized clinical trial with therapeutic application of HspB5 in relapsing-remitting multiple sclerosis patients, using intravenous doses of HspB5 was completed in 2015. Repeated administration of the lower doses of HspB5 resulted in a progressive decline in MS lesion activity, which was not seen in the placebo group (22).

Considering the abundance of HspB5 expression in peripheral tissues and its potential therapeutic value for counteracting inflammation and supporting neuroprotection, HspB5 is a promising candidate to test in the context of infection/inflammation-induced sensorineural hearing loss.

The aim of this study was to characterize the acute inflammatory phase of the disease upon infection and evaluate whether exogenous human recombinant HspB5 could be used to protect cochlear neurosensory cells in an infant rat model of *S. pneumoniae*-induced bacterial meningitis.

## Methods and Materials

All animal experiments were approved by the experimentation committee of the canton Bern, Switzerland (license to SSL BE124/16 and BE1/18) and followed the Swiss national guidelines for the performance of animal experiments.

### HspB5

For this study, GMP-grade sterile recombinant human HSPB5 (Delta Crystallon BV, Leiden, The Netherlands), was used. Previous analysis reported 5 ng/mg (0.0005 %) *E. Coli* proteins, <0.7 EU/mg endotoxins, and <75 pg/mg bacterial DNA.

### *Streptococcus pneumoniae* Inoculum

A clinical isolate of *Streptococcus pneumoniae* (serotype 3) from a patient with bacterial meningitis had been adapted to rats through several *in vivo* passages and was prepared as previously reported (8, 34). Bacteria were cultured overnight in brain-heart infusion medium. Then the bacterial culture was diluted 1:10 in fresh medium and grown for another 5 h until the logarithmic phase was reached. The bacterial culture was centrifuged for 10 min at 3,100 g at 4°C. The pellet was re-suspended in 0.85 % NaCl followed by a second centrifugation and then diluted to the desired concentration by optical density measurements at 570 nm. Final inoculum concentration was later quantified by plating serial dilutions of the inoculum on Columbia sheep blood agar (CSBA) plates.

### Infant Rat Model of Pneumococcal Meningitis With Therapeutic Intervention by HspB5

A well-established infant rat model of experimental pneumococcal meningitis was used as previously described (14, 35). A schematic is given in **Figure 3A**. Eleven-day old Wistar rats obtained from Charles River Laboratories (Sulzfeld, Germany) were infected by a single intracisternal injection of *S. pneumoniae* (10 μl of 8.20 ± 1.8 × 10^5^ cfu/ml). Control animals received same volume of saline (0.85 % NaCl). During the acute phase of meningitis, animals were weighted and clinically scored at 0, 18, 24, and 42 h post infection (hpi), as previously described (35). Disease symptoms were recorded and scored as follows: 1 = minimal or no spontaneous motor activity, coma; 2 = unable to turn upright within 30 s; 3 = turns upright within 30 s; 4 = signs of disease in terms of weight loss and/or appearance of fur, turns upright within 5 s; 5 = normal behavior, healthy. Spontaneous mortality was documented. Animals were sacrificed if scored 2 or lower.

Punctures of the cisterna magna were performed using a 30-gauge needle to collect CSF samples at 18 hpi, 24 hpi and 42 hpi to follow the inflammatory cytokine secretion pattern. A volume of about 15 μl was collected per animal per time point. CSF samples collected at 18 hpi, when animals developed symptomatic disease, were also used to confirm meningitis and quantify bacterial titer by means of serial dilution and plating on CSBA culture plates.

Antibiotic treatment with ceftriaxone (100 mg/kg, i.p. Rocephine, Roche) was initiated 18 hpi, given twice a day for up to 5 days in long term experiments.

To assess therapeutic effect of HspB5 in pneumococcal meningitis, animals were randomized for treatment with HspB5 or control. Recombinant human HspB5 was diluted in sterile saline and rats were injected intracisternally (i.c.) with 10 μg HspB5 in 10 μl saline or intraperitoneally (i.p.) with 50 μg HspB5 in 250 μl saline. These doses were chosen based on literature where they have been shown to be effective in a model of experimental stroke [50 μg i.p. daily for up to 1 week (25)], experimental autoimmune encephalomyelitis and spinal cord injury when delivered intravenously [10 μg i.v. every other day for 3 weeks; (21, 25, 36)]. For i.c. injections, HspB5 therapy was initiated shortly prior to infection (2 h before) to avoid excessive intracranial pressure by delivering inappropriate volumes of recombinant protein and bacteria concomitantly, followed by consecutive applications. This was done after CSF extraction at 18 hpi, and subsequently 3 days post infection (dpi) and 5 dpi. Control animals were injected with an equal volume of BSA in saline (10 μl of 1 mg/ml). For i.p. administration two different protocols were tested with animals receiving HspB5 or saline immediately prior to infection (2 h before), followed by consecutive applications at 18 hpi, 3 dpi and 5 dpi, or receiving HspB5 at 18 hpi, 3 dpi and 5 dpi. The examiner was blinded regarding treatment groups for the entire experiment as well as during data analyses.

Animals were sacrificed at two different time points for histological analyses. Namely at 42 hpi, to investigate the inflammatory response in the cochlea during the acute phase after infection or 4 weeks post infection, to assess hair cell and spiral ganglion survival. Animals were sacrificed with an overdose of pentobarbital (15 mg/100 g), and perfused via the left ventricle with 4 % paraformaldehyde (PFA) in phosphate-buffered saline (PBS).

### Quantitative Analysis of Cytokine Expression on the CSF/Cytokine Analysis in CSF

A panel of PM-associated cytokines (TNF-α, IL-6, IL-1β, IFN-γ, and IL-10) was measured in the CSF at 18, 24 and 42 hpi by using a magnetic multiplex assay (Rat Magnetic Luminex^®^ Assay, Rat Premixed Multi-Analyte Kit, R&D Systems, Bio-Techne) on a Bio-Plex 200 station (Bio-Rad Laboratories) as previously described (8, 37). Five microliters CSF were diluted to a final volume of 50 μl and at least 50 beads per analyte were measured. Calibration curves from recombinant standards were calculated with Bio-Plex Man-ager software (version 4.1.1) using a five-parameter logistic curve fitting. For samples below the detection limit, the value of detection limit provided by the manufacturer (TNF-α, 22.1 pg/ml; IL-6, 56.0 pg/ml; IL-1β, 26.7 pg/ml; IL-10, 18.6 pg/ml; IFN-γ, 70.5 pg/ml) was multiplied by the dilution factor.

### Determination of Hearing Capacity by Auditory Brainstem Response

Hearing capacity was assessed by recording auditory brainstem responses (ABRs) to click and pure tone stimuli on both ears with the Smart EP system (Intelligent Hearing Systems, Miami, USA), as previously described (8, 34). Animals were anesthetized with isoflurane (5 % induction, 2 % maintenance) using the Combi-Vet Vaporizer System equipped with a digital flowmeter (Rothacher Medical, Switzerland). One hundred-microsecond click stimuli and 5-ms pure tone pips (Blackman envelope; polarity alternating) were presented at a rate of 21.1 s^−1^, ranging from 100 to 20 dB SPL in 10 dB decrements (5 dB decrements close to threshold). Responses were measured at 4, 8, 16 and 32 kHz. A total of 1,024 responses were averaged at each sound level and filtered between 100 and 1,500 Hz. The hearing threshold was defined as the lowest intensity that induced the appearance of a visually detectable first peak in the recording. Later peaks were not taken in consideration for the evaluation of the threshold as they were not reliable, especially in infected animals.

### Histological Analyses

#### Cryosections

Cochleae were dissected immediately after perfusion of animals and placed for 4 h in 4 % PFA at 4°C and subsequently decalcified with Osteosoft (Merck, Germany) for at least 10 days.

Cochleae were dehydrated stepwise with 15 % for 2 h and 30 % sucrose overnight. Samples were embedded in optimal cutting temperature medium (O.C.T., Sakura, Netherlands) and cut with a cryostat (Leica CM3050 S) in 16 μm thick mid-modiolar sections, where every second section was mounted on Superfrost Plus microscopy slides (Thermo Fisher Scientific, USA) for quantification studies. To stain the samples, slides were put in a Shandon Sequenza staining rack (Thermo Fisher Scientific, USA). Sections were permeabilized for 10 min with 0.1 % Triton X-100 and blocked with blocking solution (2 % BSA, 0.01 % Triton X-100 in PBS) for 1 h at room temperature. Samples were incubated with primary antibodies (Table 1) overnight at 4°C. The next day slides were rinsed and incubated with the corresponding secondary antibody (Table 1) for 2 h at room temperature, rinsed again and mounted with a coverslip using Fluoroshield containing DAPI (Sigma).

**Table 1:** Antibodies used in the study.

	**Species**	**Dilution**	**Company**
**PRIMARY ANTIBODY**			
Anti-B-iII Tublin (TUJ)	Mouse	1:500	R & D Systems
Anti-Sox2	Mouse	1:200	RD Bioscience, USA
Anti-Sox10	Mouse	1:200	Santa Cruz Biotechnology,USA
Anti-myeloperoxidase (MPO)	Mouse	1:500	abcam
Anti-CD68	Mouse	1:500	Serotec
Anti-CD11b/OX42	Mouse	1:500	BD Bioscience, USA
Anti-Pou4f3/Brn-3c	Mouse	1:500	Santa Cruz Biotechnology,USA
Anti-alpha B-crystalline	Rabbit	1:500	Abcam, UK
Anti-iba1	Rabbit	1:500	Wako
Anti-CD206	Rabbit	1:500	Abcam, UK
Anti-Cleaved Caspase 3	Rabbit	1:500	Cell Signaling
Anti-Myo7a	Rabbit	1:500	Enzo Life Science
Anti-neutrophil elastase (M-18)	goat	1:500	Santa Cruz Biotechnology,USA
**SECONDARY ANTIBODY**			
Anti-goat AF488	Donkey	1:500	Molecular Probes
Anti-mouse Cy3	Donkey	1:500	Jackson (Milan, AG)
Anti-mouse Cy5	Goat	1:500	Jackson (Milan, AG)
Anti-mouse AF555	Goat	1:500	Invitrogen, USA
Anti-rabbit AF 647	Goat	1:500	Invitrogen, USA

Slides were visualized with a Nikon Eclipse Ti-E using a Nikon-PLAN Fluor 4x/0.13 NA, 10x/0.3 NA, 20x/0.50 NA or 40x/1.00 NA Oil objective and by a Zeiss 710 laser scanning microscope using a Zeiss Plan-Apochromat and 40x/1.3 NA oil objective for hair cell quantification.

#### SYTO9 Staining

SYTO9 (3.34 mM) and Propidium iodide (20 mM) of the LIVE/DEAD BactLight Bacterial Viability Kit (Molecular Probes) were mixed 1:1 and directly put on cochlear cryosections and incubated for 15 min at room temperature protected from light. Sections were washed once with PBS and mounted with Fluoroshied containing DAPI (Sigma) and visualized with a Nikon Eclipse Ti-E using a Nikon-PLAN Fluor 40x/1.00 NA Oil objective.

#### Sirius Red Staining

Cryosections of cochleae at 1 month post infection were immersed in xylene for 10 min at room temperature and transferred to ethanol (100, 100, 80, and 70%, all 10 s) before stained by using 0.1 % solution of Picro Sirius Red solution (Sigma) for 1 h at room temperature. Subsequently, sections were rinsed in 0.01 N HCl for 2 min. Sections were dehydrated in ascending concentrations of ethanol (70, 80, 100, and 100%, each 10 s) and cleared in two stages in xylene, 10 min each. Sections were mounted with Eukitt quick-hardening mounting medium (Fluka Analytical).

#### TUNEL

TUNEL assay was performed by automated staining using Bond RX (Leica Biosystems) immunostainer. Paraffin slides were dewaxed in Bond dewax solution (product code AR9222, Leica Biosystems). Antigen retrieval was performed with Protease incubation for 25 min at 37°C. TdT enzyme incubation was performed for 20 min with antibody diluent (Leica AR9352), TdT Buffer (Promega M1893), DIG-11-dUTP (Roche, Ref 11570013910) and TdT Enzyme (Promega M1875) followed by incubation with mouse anti DIG-FITC (1:500) for 15 min and Rabbit anti FITC (1:1000) for 15 min. The reaction was visualized using AP (Alkaline phosphatase)-rabbit polymer for 15 min and fast red as red chromogen (Red polymer refine Detection, Leica Biosystems, Ref DS9390). Finally, the samples were counterstained with Haematoxylin and mounted with Aquatex (Merck). Stainings were performed by the translational research unit of the institute of Pathology, University of Bern.

#### Preparation of Organ of Corti Whole Mounts of Adult Rats and Immunofluorescent Staining

The fixed and decalcified cochleae were dissected with forceps (World Precision Instruments) and sapphire blades (World Precision Instruments) using a Nikon SMZ800 binocular microscope. The cochlea was cut in half, longitudinal from the apex to the oval window through the modiolus, the apical turn was left as one piece. Basal, middle and apical turns were separated and hook was cut off the lowest part of the basal turn resulting in a total of five pieces per cochlea. The organ of Corti was isolated by removing the lateral wall and spiral ganglion.

The tissue pieces were permeabilized with 3 % Triton X-100 for 30 min, rinsed three times with PBS, and incubated in blocking solution for 2 h at room temperature. The hair cell-specific antibody against Myosin7a was incubated for at least 12 h at 4°C. The tissue was rinsed three times with PBS and incubated with the secondary antibodies and Phalloidin ATTO488 (Sigma) diluted 1:500 in blocking solution overnight at 4°C. Each tissue piece was rinsed with PBS and incubated for 15 min with 1:1000 DAPI at room temperature before being rinsed again and transfer to a 24 well glass bottom plate (Corning, USA) for confocal imaging.

Image acquisition was performed with a Zeiss 710 laser scanning microscope using a Zeiss Plan-Apochromat 10x/ 0.3 NA and 20x/0.8 NA objective for hair cell quantification.

### Preparation of Organ of Corti Organotypic Cultures and Exposure to *S. pneumoniae*

Cochlear explants were isolated as previously reported (38). The organ of Corti from postnatal day 2 (p2) Wistar rats was plated on a Transwell insert (6-well format, Corning, USA) with a permeable polyester membrane (0.4 μm pore size). Membranes were pre-coated with Cell-Tak (Corning, USA) according to manufacturer's protocol. Explants were cultured on 1.5 ml DMEM/F12 (10 % FBS, 0.01 % Ampicillin) that was added to the lower compartment under the insert overnight at 37 °C with 5 % CO_2_ before any treatment.

To mimic the *in vivo* conditions, *S. pneumoniae* were added to the medium in the lower compartment. The inoculum concentration was chosen according to the expected bacterial titers after bacterial proliferation in the CSF *in vivo* during the acute phase (18 hpi) of PM, i.e., 1.7 × 10^8^ cfu/ml. Bacteria were prepared as described above and finally re-suspended in ampicillin- and FBS-free otic medium. Organ of Corti were exposed to bacteria for 2 h, washed and cultured for 4 more days in full otic medium (39). To assess the effect of HspB5, recombinant human HspB5 (50 μg/ml) was added to the cultures during exposure to bacteria and the following 4 days of cell culture.

### Immunofluorescent Staining of Explants

At the end of the experiment explants were fixed with 4 % PFA for 10 min, washed and stored in PBS at 4°C until staining. The insert membrane was cut and explants were transferred to a 24 well plate. Explants were permeabilized for 30 min with 3 % Triton X-100 (Sigma-Aldrich, USA) and blocked for 1 h with blocking buffer (2 % BSA, 0.01 % Triton X-100 in PBS) at room temperature. Explants were incubated with the hair cell specific anti-Pou4f3 antibody, the supp-orting cell marker anti-Sox2 antibody and HspB5 specific antibody in blocking buffer overnight at 4°C. On the following day, tissue was rinsed three times and incubated with the secondary antibodies at room temperature for 2 h. Finally, samples were rinsed again three times with PBS and mounted on a glass slide with Fluoroshield containing DAPI (Sigma, USA). Image acquisition was performed with a Zeiss 710 laser scanning microscope using a Zeiss Plan-Apochromat and 20x/0.8 NA objective for hair cell quantification.

### Data Analysis of Histological Samples

Image processing was performed with the open source image processing software FIJI, version 2.0.

*Neutrophil extracellular traps (NETs) quantification*: Five non-consecutive mid-modiolar cryosections per cochlea were used to quantify the area of the NETs (stained by MPO) in the scala tympani. The occluded area by NET formation was measured and calculated as percentage of the area of scala tympani.

*Spiral ganglion neuron quantification:* The density of spiral ganglion neurons was calculated on five non-consecutive mid-modiolar sections by counting βIII-Tubulin positive cells normalized to the area of the Rosenthal's canal.

*Hair cell quantification*: Pou4f3 or Myo7a positive cells of the organ of Corti were counted at three random microscopic fields for each cochlear region (basal, middle, apical) covering in total at least 1,200 μm. The number of hair cells (IHCs and OHCs) was expressed as unit per 100 μm.

### Statistical Analysis

Statistical analysis was performed with GraphPad Prism software (Prism 7 for Windows; GraphPad Software Inc., San Diego, CA). Normal distribution of the datasets was tested by D'Agostino & Pearson and KS normality test. One-way ANOVA with Tukey's multiple comparison test was used to evaluate whether the means of more than two populations differ. Two-way ANOVA was used to determine the interaction of two independent variables (e.g., location in cochlea and treatment) on a dependent variable (e.g., hair cell count). Unpaired *t*-tests were used to compare parametric data sets of two groups. Welch's correction was applied if variances were not equal (F test).

If not stated otherwise, results are presented as mean values ± standard deviations. In box-plots, the horizontal line within the box represents the median, the cross indicates the mean, the top and the bottom of the box mark the 75th and 25th percentiles; the whiskers represent the range of the data (min-max). A two-tailed *p* < 0.05 was considered statistically significant, with *p* < 0.05 (^*^), *p* < 0.01 (^**^), *p* < 0.001 (^***^) and *p* < 0.0001 (^****^).

## Results

### Pneumococcal Meningitis Induces Inflammatory Cell Infiltration to the Cochlea

We initially assessed the inflammatory response within the cochlea as a consequence of bacterial meningitis. We analyzed the presence of macrophages and neutrophils 42 h post infection, in the acute phase of the disease, using immunostaining of mid-modiolar sections.

**Figure 1:**
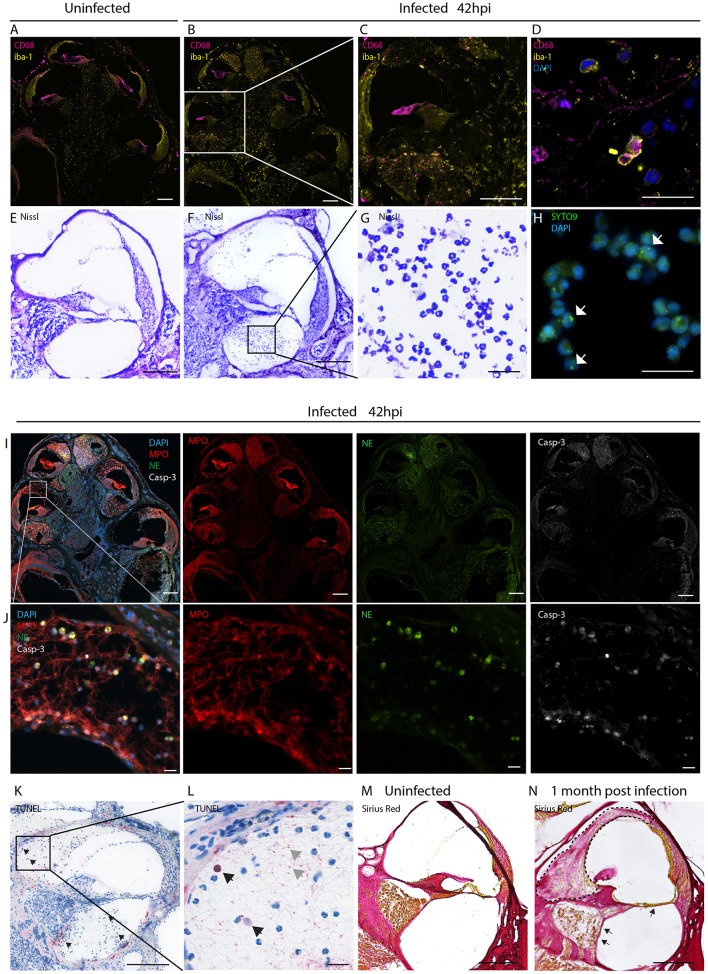
Inflammatory infiltration of the perilymphatic spaces. Immunostainings for macrophages markers CD68 and iba1 on mid-modiolar sections of rat cochlea **(A)** uninfected and **(B)** 42 h post infection with *S. pneumonia*. **(C)** Close up of a middle turn and **(D)** of the perilymphatic space. **(E)** Nissl staining of a cochlear middle turn of an **(E)** uninfected animal and **(F)** 42 h post infection. **(G)** Higher magnification of cells infiltrating in the perilymphatic space showing the characteristic segmented nuclei morphology of neutrophils. **(H)** Presence of phagocytized *S. pneumoniae* stained with SYTO9 (white arrows) in infiltrating leukocytes. **(I)** Overview of mid-modiolar cryosections 42 h post infection stained for myeloperoxidase (MPO) and neutrophil elastase (NE) and cleaved Caspase-3 (Casp-3). Merge and single channels are shown **(J)** close-up view of the perilymphatic spaces (scala vestibuli) with reticular formed neutrophil extracellular traps (NETs). **(K)** TUNEL staining of a cochlear middle turn, 42 h post infection. **(L)** close-up of scala vestibuli. Black arrows indicate positive cells, gray arrows, extracellular DNA. **(M)** Collagen staining of an uninfected animal and **(N)** an animal 1 month post infection. Fibrosis, detected by sirius staining, is indicated by the dashed line. Arrows highlight loss of the organ of Corti structures and of the spiral ganglion neurons. Scale bars in overview images and middle turns indicate 200 μm while scale bars of **(D,G,H,J,L)** magnified perilymphatic spaces represent 20 μm.

In uninfected conditions, resident macrophages were present in various locations of the cochlea. CD68+ macrophages were most prominently found in the modiolus and the spiral ganglion (Figure 1A; Figures S1A–C). CD206+ macrophages were additionally seen in the spiral ligament (Figures S1G–L). Iba1+ macrophages were numerously present in the modiolus, the spiral ganglion, in the spiral ligament and in stria vascularis (Figure 1A; Figures S1A-C).

During the acute phase of the infection, macrophages infiltrated the perilymphatic spaces in the cochlea adopting round morphology (Figures 1B–D). Co-expression of Iba1 with CD68 and expression of CD11b (Figures S1 D-F, M-O), were indicative of cell activation.

Moreover, we observed infiltration of numerous granulocytes in the perilymphatic spaces (Figures 1E–J). Nissl staining showed that many of the infiltrating cells displayed segmented nuclei, typical of neutrophils (Figures 1F,G). 42 h post infection SYTO9 staining revealed the presence of phagocytized *S. pneumoniae* within the infiltrating leukocytes (Figure 1H).

Myeloperoxidase and neutrophil elastase staining confirmed presence of neutrophils releasing reticular-shaped neutrophil extracellular traps (NETs) (40) (Figures 1I,J). Concomitantly, we observed apoptotic cell death of the infiltrating cells, detected by cleaved caspase 3 (Figures 1I,J) and TUNEL staining (Figures 1K,L). The latter also revealed extracellular DNA in the perilymphatic spaces, possibly being NET-DNA (41, 42).

When cochlear histology was performed 4 weeks post infection, in 21 % of the cases (4/19), infiltrating granulocytes were replaced by fibrotic occlusions in the scalae vestibuli and tympani (Figure 1N, dashed line) ranging from slight occlusion to almost complete occlusion of the perilymphatic space (sc. vestibuli: 18.3 to 100 %, sc. tympani: 5.6 to 72.9 %). A representative image of fibrotic occlusion of the scala vestibuli is shown in Figure 1N next to an uninfected animal with clear cochlear ducts in Figure 1M. In severe cases the organ of Corti was degenerated and spiral ganglion neurons were lost (Figure 1N, arrows).

No inflammatory cells and no occlusions were found the endolymphatic space.

### Local Administration of HspB5 to the CSF Space Showed Anti-inflammatory Effects

**Figure 2:**
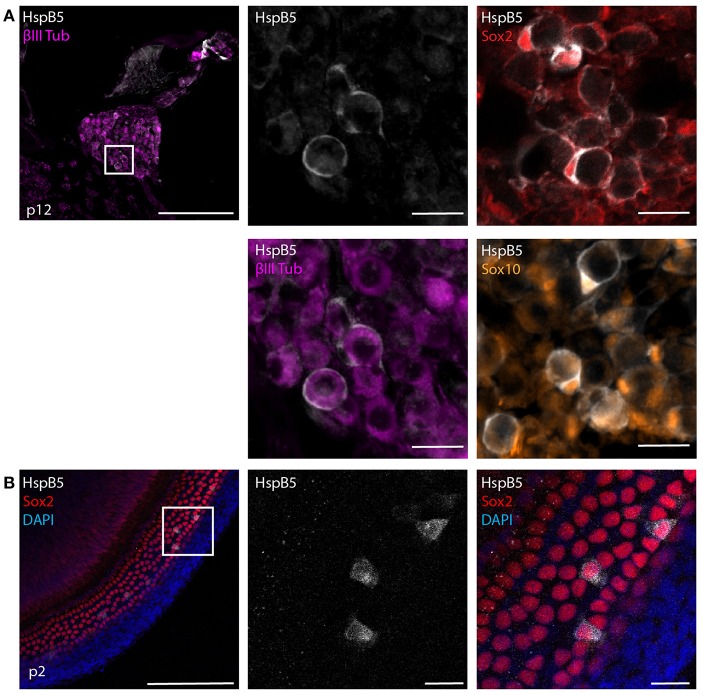
Endogenous expression of HspB5. **(A)** Immunostaining of mid-modiolar cryosection stained for HspB5 (white) and the Schwann cells markers Sox2+ (red) and Sox10+ (orange) or the neuronal marker βIII Tubulin (magenta) in p12 rats. **(B)** Immunostaining of organ of Corti whole mounts of p2 animals for HspB5 (white) and the supporting cells marker Sox2 (red). Scale bars in overview images indicate 200 μm while scale bars of magnified areas represent 20 μm.

HspB5 is endogenously expressed in different cell types, including oligodendrocytes of the CNS (21, 23) and lack of this protein results in higher susceptibility to inflammatory damage (21, 25). We first analyzed the endogenous localization of HspB5 in the cochlea of uninfected rats. At 12 days of age, a clear expression of HspB5 was observed in a subset of Sox2+/Sox10+ Schwann cells in the Rosenthals' canal (Figure 2A) surrounding the TUJ+ spiral ganglion neurons, and also in some sparse cells in spiral ligament and Reissner's membrane (data not shown). In contrast, we could not detect HspB5 within the sensory epithelium, neither in 12 days old nor at 6 weeks of age (data not shown), while some supporting cells in the organ of Corti displayed positivity in very young postnatal animals at p2 (Figure 2B).

**Figure 3:**
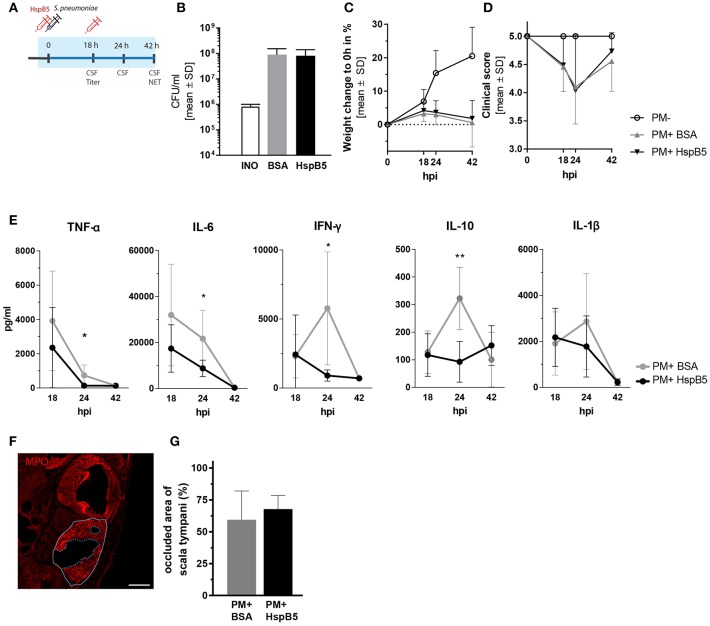
Intracisternal administration of HspB5 and effects during the acute phase of pneumococcal meningitis. **(A)** Schematic representation of the experimental setup to study the acute phase outcomes. Intracisternal delivery of HspB5 (red syringe) is performed immediately prior to the infection and at 18hpi. Antibiotic treatment is initiated at 18 hpi and administered i.p. The acute phase (0–42 h) is indicated in blue. **(B)** Bacterial titer at 18 hpi indicates colony forming units in 1 ml of CSF **(C)** Relative weight change during acute infection showed a slight increase within the first 18 hpi and weight loss with the onset of clinical symptoms and antibiotic treatment. **(D)** Clinical scores deteriorated during the first 24 h after infection before sick animals recovered. **(E)** Quantification of inflammatory cytokines in the CSF at 18hpi, 24hpi, and 42hpi of untreated (gray, *n* = 9 animal) and treated animals (black, *n* = 10 animals) Statistical differences were assessed using unpaired *t*-test with Welch correction ^*^p < 0.05; ^**^p < 0.01. **(F)** Representative immunofluorescence of NET occlusion (basal turn) in infected animals with delineated areas of the scala tympani (white line) and NET occlusion (blue dashed line). Scale bar = 200 μm. **(G)** The ratio between the NET-occupied area and the area of scala tympani 42 h post infection, is plotted for untreated (gray, *n* = 7 animals) and HspB5-treated (black, *n* = 4 animals).

We then tested the effects of HspB5 administration on the inflammatory process caused by pneumococcal meningitis. In order to obtain high local concentrations of HspB5 in the CSF and perilymphatic spaces during the acute phase of inflammation, the recombinant protein was delivered directly to the cisterna magna immediately prior to the infection and subsequently at 18 h post infection (Figure 3A). The control group received an equivalent amount of bovine serum albumin (BSA). Intracisternal infection was performed by injection of 10 μl saline containing 8.20 ± 1.8 × 10^5^ cfu/ml living *S. pneumoniae*. This concentration of the inoculum was previously shown to cause moderate-to-severe damage to hair cells and spiral ganglion neurons (8).

The infection resulted in similar CSF bacterial concentration in the control (8.98 ± 6.38 × 10^7^ cfu/ml, *n* = 10 animals) and the HspB5-treated group (8.16 ± 5.88 × 10^7^ cfu/ml, *n* = 8) 18 h post infection (Figure 3B). Infected animals showed reduced weight gain (Figure 3C) and worsening of the clinical score compared to mock-infected animals (*p* < 0.0001) (Figure 3D). No significant difference in bacterial titer, weight change and clinical score was seen between the two treatment groups (BSA vs. HspB5). After repeated treatment with ceftriaxone, animals recovered from the infection. Clinical conditions improved and weight gain was normalized.

The CSF, collected at different time points after infection (18 hpi, 24 hpi, and 42 hpi), was analyzed for inflammatory cytokine secretion. Bacterial infection induced a rapid increase in cytokine production. HspB5-treated animals had significantly reduced levels of TNF-α (*p* < 0.05), IL-6 (*p* < 0.05), IL-10 (*p* < 0.01), and IFN-γ (*p* < 0.05) at the peak of the inflammatory process (24 hpi, Figure 3E).

When we assessed the presence of NETs at 42 hpi, we observed up to 90 % of the area of scala tympani occluded by NETs (Figure 3F) but we could not detect any differences as a result of HspB5 treatment (Figure 3G).

### Intracisternal Administration of HspB5 Reduced Hearing Loss

**Figure 4:**
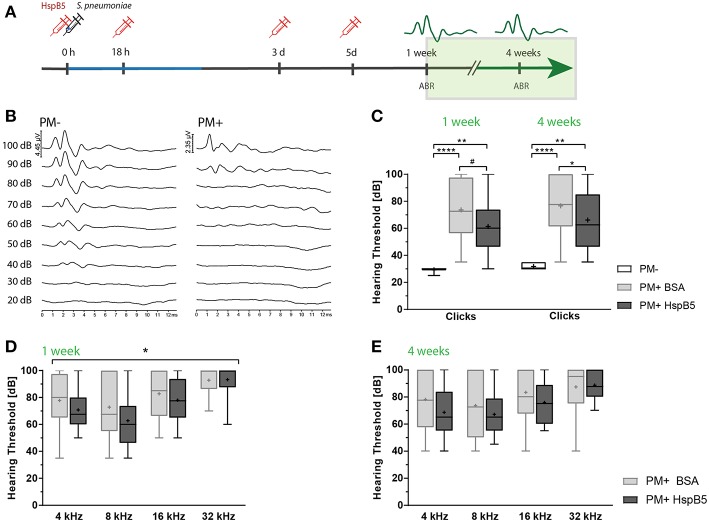
Intracisternal administration of HspB5 results in improved hearing thresholds. **(A)** Schematic representation of the experimental setup to study late phase outcomes. Intracisternal delivery of HspB5 (red syringe) is performed immediately prior to the infection and at 18hpi, 3 days, and 5 days post infection. **(B)** Representative example of hearing thresholds of a control animal (PM-: 30 dB, left) and an infected animal (PM+: 90 dB, right). **(C)** ABR recording using broad band click stimuli 1 and 4 weeks post infection in uninfected (*n* = 6 ears of 3 animals) and infected animals [untreated: (*n* = 20 ears/10 animals) and HspB5 treated: (*n* = 20 ears/10 animals)]. **(D)** Pure tone ABR thresholds 1 and **(E)** 4 week post infection for untreated and HspB5 treated animals (*n* = 20 ears/10animals per group). Box and whisker plot quartiles from min-max. Statistical differences were assessed by one-way ANOVA with Tukey's multiple comparison test. ^#^*p* value for unpaired *t*-tests *p* = 0.053 (in **C**) and two-way ANOVA (in **D,E**). ^*^p < 0.05; ^**^p < 0.01 ^****^p < 0.0001.

Hearing tests were performed at 1 and 4 weeks post infection by assessing auditory brainstem responses (ABR) (Figure 4A). Representative click-ABR recordings are shown in Figure 4B. Rats were presented to a broadband click stimulus and pure tones at 4, 8, 16, and 32 kHz. Hearing thresholds for clicks were significantly elevated at 1 week post infection and persisted until 4 weeks post infection compared to the mock-infected controls (*p* < 0.0001) (Figure 4C).

Intracisternal HspB5-therapy significantly improved hearing thresholds. We saw a trend for improved hearing thresholds of broad band clicks compared to the untreated group already 1 week post infection (61.25 dB ± 18.63 vs. 73.5 dB ± 20.2, *p* = 0.053, unpaired *t*-test between the two infected groups, *n* = 10 animals per group; *p* = 0.0978 one-way ANOVA with Tukey's multiple comparison test, when including the control group.) and the difference was statistically significant at 4 weeks post infection (61.25 dB ± 61.25 vs. 76.5 dB ± 21.95, *p* = 0.0230, unpaired *t*-test, *n* = 10 animals per group; *p* = 0.0406, one-way ANOVA with Tukey's multiple comparison test) (Figure 4C). Pure tones ABR thresholds were lower at all frequencies tested in the HspB5-treated group, showing statistically significant differences at 1 week post infection (two-way ANOVA, *p* = 0.0409) (Figure 4D), while did not reach statistical significance at 4 weeks (Figure 4E).

### Local HspB5 Delivery Protects Against Meningitis-Induced Sensory Cell Loss

**Figure 5:**
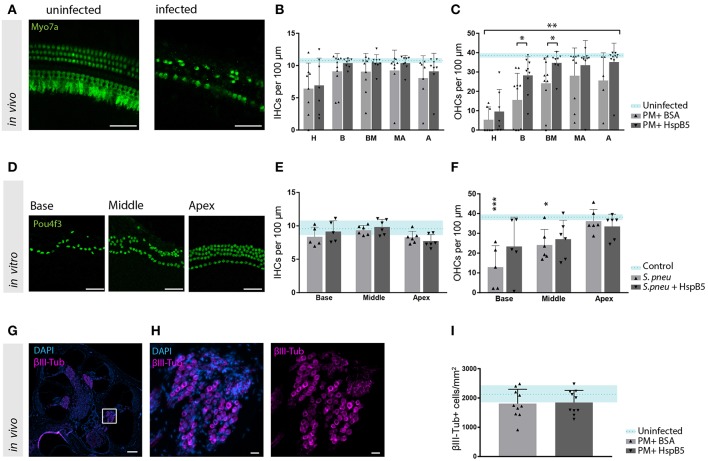
HspB5 protects against *S. pneumonia* -induced sensory hair cell loss. **(A)** Representative example of a whole mount preparation of the organ of Corti stained with hair cells specific Myo7a (green), scale bar = 50 μm **(B)** Quantification of inner hair cells and **(C)** outer hair cells at 4 weeks post infection, uninfected animals (*n* = 3) indicated in blue as mean (blue dashed line) and standard deviations (blue shade), hair cell count of infected animals (*n* = 10/group) represented in bar graphs. 7–10 whole mount preparations were quantified per cochlea; H, hook; B, base; BM, base-middle; MA, middle-apex; A, apex. **(D)** Representative confocal images of the organ of Corti immunostained for hair cells (Pou4f3, green) 4 days after exposure to *S. pneumonia*. Basal, middle and apical turns are shown, scale bar = 50 μm **(E)** quantification of inner and **(F)** outer hair cells *in vitro* without bacteria *n* = 4 (light blue); untreated (gray) *n* = 6 and HspB5-treated (black) *n* = 6. **(G)** Mid-modiolar cross section of a control rat cochlea immunostained for the neuronal marker βIII-Tubulin (magenta) and cell nuclei (DAPI, blue), scale bar = 200 μm. **(H)** The Rosenthal's canal in the basal turn is shown at higher magnification, scale bar = 20 μm **(I)** Neuronal density quantification in the basal turn in uninfected (*n* = 3), infected untreated or HspB5-tretaed (*n* = 10 animals per group). Means with SD are indicted in the bar graphs. Dashed line with shade represents average count of uninfected cochlea with SD. A two-way ANOVA was used for determining interaction between variables and an unpaired *t*-test with Welch's correction was used for single comparison between untreated and HspB5 treated (in **B** and **C**) ^*^p < 0.05; ^**^p < 0.01. A one-way ANOVA with Tukey's multiple comparison test was used to evaluate whether the means of the population s differ in **(E,F)** ^*^p < 0.05; ^***^p < 0.001.

Hair cell quantification was then performed on whole mount preparation of the organ of Corti at 4 weeks after infection (Figure 5A). We observed a moderate inner hair cell (IHC) loss, which was most pronounced in the hook, but no differences between the treatment groups were observed (Figure 5B). Outer hair cells (OHC) were instead strongly affected, especially in the basal and mid-basal part of the organ of Corti (p < 0.05, t-test). A higher number of outer hair cells was still intact 4 weeks after infection in the HspB5-treated group (two-way ANOVA, *p* = 0.0015). Interestingly, we observed statistically significant differences in the basal and mid-basal portions (Figure 5C).

To further confirm the protective effect of HspB5 on the survival of hair cells to pathogen-induced damage, we performed *in vitro* experiments using organotypic cultures of the organ of Corti. Exposure of the organ of Corti explants to bacteria for 2 h caused OHC loss, more pronounced toward the base (Figures 5D,F). No significant changes were observed for IHC (Figure 5E). Outer hair cells were significantly lost at the base and middle turns compared to the unexposed samples (base: 12.94 OHC ± 10.83 vs. 37.31 OHC ± 5.23, middle: 24.08 OHC ± 7.95 vs. 38.39 OHC ± 3.375, p < 0.05). When HspB5 (50 μg/ml) was added to the culture medium during exposure to the bacteria and for the following 4 days, OHCs loss in the base and middle portion was no longer statistically significantly different from the untreated explants (Figure 5F).

Given the previously reported neuroprotective effect of HspB5 as well as its endogenous localization in the Rosenthals' canal, we also analyzed the survival of spiral ganglion neurons (Figures 5G,H). We focused specifically in the basal portion of the cochlea as we had previously reported a more significant damage (8). The large majority of animals tested in these experiments showed however only minimal loss of spiral ganglion neurons after meningitis in this region (Figure 5I), whereas middle and apical region appeared undamaged. This did not allow to assess the putative protective effects of HspB5 effectively.

### Systemic Administration of HspB5 Had No Effect on Pro-inflammatory Cytokine Profile, Hair Cell Survival, and Hearing Threshold

We additionally assessed whether HspB5 exerted effects when administered systemically. Animals received either saline or HspB5 intraperitoneally. Moreover, we used a paradigm more close to clinical practice, where adjuvant treatment is initiated concomitant to antibiotic therapy starting 18 hpi, when first clinical signs appeared. Further HspB5 i.p. doses were applied at day 3 and 5, as previously described in Figure 4A. Animals were sacrificed either during the acute phase of the disease (42 hpi), or 1 month after infection, after assessment of hearing thresholds.

**Figure 6:**
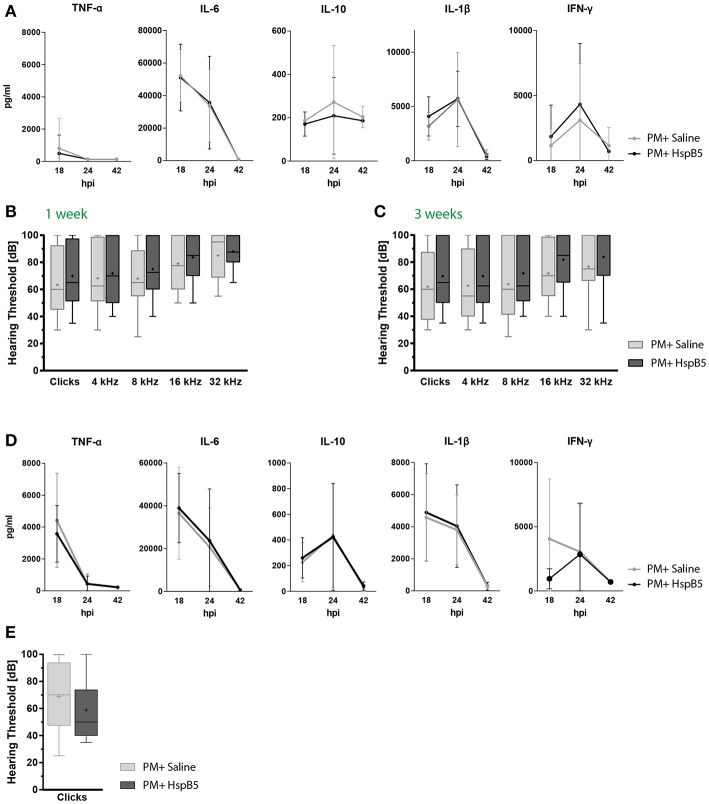
Intraperitoneal administration of HspB5 does not change inflammatory cytokine profiles nor hearing thresholds. **(A)** Animals received a single HspB5 i.p. injection at 18hpi. Quantification of inflammatory cytokines in the CSF at 18, 24, and 42 h post infection; untreated (*n* = 9, gray) and HspB5-treated (*n* = 10, black) **(B)** Animals received a HspB5 i.p. injections at 18hpi, 3 days, and 5 days post infection. ABR hearing thresholds for clicks and pure tones 1 week and **(C)** 3 weeks post infection; untreated (*n* = 26 ears/13 animals, gray) and HspB5-treated (*n* = 26 ears/13 animals, black). **(D)** Animals received i.p. injections 2 h prior to infection, as well as at 18 hpi. Quantification of inflammatory cytokines in the CSF at 18, 24, and 42 h post infection; untreated (*n* = 11, gray) and HspB5-treated (*n* = 10, black) **(E)** Animals received i.p. injections 2 h prior to infection, as well as at 18 hpi, 3 days, and 5 days post infection. ABR hearing thresholds for clicks 4 weeks post infection; (*n* = 28 ears/14 animals, gray) and HspB5-treated (*n* = 24 ears/12 animals black). Box and whisker plot quartiles from min-max. Graphs show mean values with standard deviation. Statistical differences were assessed by single comparison using unpaired *t*-test and two-way ANOVA.

In this paradigm, we did not see a difference between the treated and untreated animals, when animals were injected i.p., neither in pro-inflammatory cytokine secretion (Figure 6A), nor for hearing thresholds (Figures 6B,C). NET formation, spiral ganglion neuron density, hair cell number and presynaptic ribbons counts were also not significantly influenced by the treatment at 1 month after infection (data not shown).

We reasoned HspB5 may have reached the CSF and cochlear fluid too late compared to the inflammatory mediators using this treatment protocol, explaining its inefficacy. Therefore, we assessed HspB5- i.p. treatment in an additional cohort of animals, where administration started immediately prior to bacterial infection, and further delivered at 18 hpi, 3 dpi, and 5 dpi, as done for the i.c. administration. Also in this case however, we did not detect a difference in cytokine production (Figure 6D). A trend to improved ABR hearing thresholds was observed for broad-band clicks in the treated group at 4 weeks post infection, which however did not reach statistical significance (Figure 6E).

These results suggest that systemic delivery may not allow for reaching a sufficient concentration of HspB5 in the CSF and perilymphatic space to exert a protective effect in the acute phase of the disease.

## Discussion

The previously reported anti-inflammatory, anti-apoptotic and neuroprotective effect of the small heat shock protein HspB5 (21, 30, 36, 43, 44) prompted us to examine whether HspB5 could be used as a therapeutic agent to counteract bacterial meningitis-induced damage to the inner ear.

Our results demonstrate that intracisternal HspB5 therapy results in an increased outer hair cell survival and improved hearing thresholds after pneumococcal infection. This is the first report highlighting the use of HspB5 as a potential treatment option for bacterial meningitis-induced hearing loss.

We tested two routes of administration of the recombinant protein: local and systemic. Delivery to the inner ear via the round window niche was not feasible because animals suffering from pneumococcal meningitis are in too poor health conditions to support prolonged surgical procedures under anesthesia during the acute phase of the disease. We therefore decided to deliver the recombinant protein through i.c. injection, directly into the CSF, a procedure that does not require anesthesia and is performed very rapidly (less than a minute) in the present model. Intracisternal injection allows for direct access to the perilymphatic fluids, using similar routes as used by bacteria, though the cochlear aqueduct. While i.c. delivery allows direct access to the inner ear, the anti-inflammatory activity could also be the result of a modulation of the immune response exerted in the meningeal spaces and in the central nervous system, for example by binding to and modulating the activity of microglial cells in the brain parenchyma, as shown in other models (31). This would result consequently in less inflammatory products reaching the inner ear. Alternatively, or in combination with the above, local effects could be exerted through interaction with microglia and leukocytes in the cochlea and other inner ear compartments, or directly with cells within the sensory epithelium. This is also supported by the experiments conducted *in vitro*, where we have been able to detect hair cell protection against *S. pneumoniae*-induced damage, when applying HspB5 to the culture medium.

Local administration of HspB5 reduced inflammatory cytokine production, namely TNF-α, IL-6, and INF-γ, which are well-known to induce secondary inflammatory responses, including leukocyte infiltration and scar formation (45). It has already been reported that reduced inflammatory cytokine production in this model of pneumococcal meningitis is associated with reduced neuronal injury (34, 46, 47). Furthermore, inflammatory mediators are directly implicated in cochlear damage. For instance, TNF-α has been shown to trigger caspase expression or activation, leading to apoptosis (48). *In vitro*, excessive level of TNF-α increased the expression of apoptosis-related genes and induced cochlear cell death of outer hair cells in OC explant (49, 50). *In vivo*, anti-inflammatory strategies using for example TNFα-antibodies (51) or reducing the amount of TNF-α in the CSF via inhibition of the TNF-α-converting enzyme (TACE) with doxycycline (52), improved hearing function and reduced cochlear injury in the context of meningitis (51). In the present study, we detected reduced concentrations of TNF-α during bacterial meningitis in animals treated with HspB5 and observed attenuated hearing loss with better hair cell survival after recovery from infection in the these animals compared to the untreated cohort.

Interleukin-10 induction instead has been previously observed exclusively in severely infected animals (8), suggesting a secondary anti-inflammatory response. HspB5 dampening of IL-10 levels may correlate with an overall reduced inflammation.

Neutrophil activity, assessed here by NET formation in the perilymphatic space was however not sensitive to HspB5. The role of NETs, as well as all other inflammatory processes required for bacterial clearance and resolution of the infection, are nevertheless dual and while implicated in the development of the pathology, they are needed to protect against infections (53, 54). We have also attempted to quantify the numbers of macrophages infiltrating in the cochlea in response to meningitis and in the two treatment groups. We specifically focused on Iba1+ cells in the Rosenthal's canal. While we did not observe differences between the HspB5 and the saline-treated groups (data not shown), the branched morphology of the cells does not allow for a precise assessment using tissue sections. Alternative imaging modalities of entire cochlea whole mounts (7) would need to be optimized in order to adequately address this question.

In PM, where the damage is induced by an acute inflammatory reaction, limited to approximately 40 h, the time window for action of an effective anti-inflammatory therapy is limited. Reaching sufficiently high local concentration of the compound in a timely adequate manner is therefore technically challenging. We tried to control for this aspect by delivering HspB5 therapy at the time of infection and further applied the at 18 h post infection. This should allow for a high local concentration at the time point when the cytokine burst occurs. While the i.c. delivery significantly reduced inflammatory cytokine production in this paradigm, i.p. injection using the same schedule was ineffective.

It has been reported that HspB5 is able to cross the blood brain barrier especially under inflammatory conditions (36, 55). Since pneumococcal meningitis causes increased permeability of the blood brain barrier, and the overshooting cochlear inflammation is accompanied by blood labyrinth barrier disruption, proven by increased Evans Blue extravasation (12, 56), we assumed HspB5 would cross both barriers also during pneumococcal meningitis. However, we did not observe any changes in the inflammatory reaction, cytokine production or sensorineural hearing loss phenotypes, when HspB5 was delivered systemically. The results of the systemic delivery paradigm therefore bring about the question of whether HspB5 did not reach, or did not reach in sufficient amount, or at the right time point, the inner ear and CSF since our effort to detect the i.p. delivered compound by classical immunostaining of the cochleae was unsuccessful.

In clinical practice, HspB5 could be applied directly in the CSF space, in a timely manner. Alternatively, intratympanic injection may be rather feasible in a human patient compared to infant rats. Testing the compounds in different models, involving less severe but chronic inflammation such chronic exposure to moderate levels of noise (57), may be alternatively used to better reveal the role of HspB5 in oto-protection.

In conclusion, our data provides evidence that HspB5, when administrated adequately, attenuates the inflammatory response and results in better outer hair cell survival with improved hearing thresholds. Since HspB5 was only beneficial when given directly to the CSF compartment at the time of infection, but not when delayed longer and delivered systemically, the early changes in cytokine expression observed upon HspB5 treatment may be crucial to conferring protection.

## Data Availability

All datasets generated for this study are included in the manuscript and/or the Supplementary Files.

## Ethics Statement

All animal experiments were approved by the experimentation committee of the canton Bern, Switzerland (license to SSL BE124/16 and BE1/18) and followed the Swiss national guidelines for the performance of animal experiments.

## Author Contributions

SE, MR, DG, SL, RR, and PS conceived and designed the study. SE, GF, MB, MR, and MP performed experiments. SE, GF, MR, DG, and SL analyzed the data. SE, MR, DG, JvN, and SL contributed to writing of the manuscript. All authors read and approved the final manuscript.

### Conflict of Interest Statement

RR is the CEO of Audion Therapeutics, a biotech-company developing new strategies for hearing loss. JvN worked for Delta Crystallon BV but declares no conflict/financial interests. The remaining authors declare that the research was conducted in the absence of any commercial or financial relationships that could be construed as a potential conflict of interest.

## Supplementary Material

Figure S1 Macrophages in the cochlea. **(A–C)** Mid-modialar section of uninfected rat cochlea immunostained for CD68 (magenta) and iba1 (yellow), **(D–F)** Mid-modiloar sections of rat cochlea 42 hours post infection with *S. pneumoniae* immunostained for CD68 and iba1, **(G–I)** uninfected cochleae stained for CD68 (magenta) and CD206 (gray), **(J–L)** cochleae 42 hours post infection stained for CD68 and CD206, scale bars = 20 μm. **(M)** Representative example of CD11b (yellow) immunostaining and DAPI (blue) in uninfected animals and **(N)** 42hpi, scale bars = 200 μm **(O)** detail of the cells in the perilymphatic space, scale bar = 20 μm.

## References

[1] van de Beek D de Gans J Spanjaard L Weisfelt M Reitsma JB Vermeulen M. Clinical features and prognostic factors in adults with bacterial meningitis. N Engl J Med. (2004) 351:1849–59. 10.1056/NEJMoa040845 15509818

[2] Østergaard C Konradsen HB Samuelsson S. Clinical presentation and prognostic factors of Streptococcus pneumoniae meningitis according to the focus of infection. BMC Infect Dis. (2005) 5:93. 10.1186/1471-2334-5-93 16253143 PMC1295586

[3] Hirose K Rutherford MA Warchol ME. Two cell populations participate in clearance of damaged hair cells from the sensory epithelia of the inner ear. Hear Res. (2017) 352:70–81. 10.1016/J.HEARES.2017.04.006 28526177 PMC5544544

[4] Kalinec GM Lomberk G Urrutia RA Kalinec F. Resolution of cochlear inflammation: novel target for preventing or ameliorating drug-, noise- and age-related hearing loss. Front Cell Neurosci. (2017) 11:192. 10.3389/fncel.2017.00192 28736517 PMC5500902

[5] Liu W Molnar M Garnham C Benav H Rask-Andersen H. Macrophages in the human cochlea: saviors or predators—a study using super-resolution immunohistochemistry. Front Immunol. (2018) 9:223. 10.3389/fimmu.2018.00223 29487598 PMC5816790

[6] Møller MN Brandt C Østergaard C Caye-Thomasen P. Bacterial invasion of the inner ear in association with *Pneumococcal meningitis*. Otol Neurotol. (2014) 35:e178–e186. 10.1097/MAO.0000000000000305 24569797

[7] Perin P Voigt FF Bethge P Helmchen F Pizzala R. iDISCO+ for the Study of neuroimmune architecture of the rat auditory brainstem. Front Neuroanat. (2019) 13:15. 10.3389/fnana.2019.00015 30814937 PMC6381022

[8] Perny M Roccio M Grandgirard D Solyga M Senn P Leib SL. The severity of infection determines the localization of damage and extent of sensorineural hearing loss in experimental pneumococcal meningitis. J Neurosci. (2016) 36:7740–9. 10.1523/JNEUROSCI.0554-16.2016 27445150 PMC6705551

[9] Täuber MG Moser B State-of-the-art clinical article cytokines and chemokines in meningeal inflammation : biology and clinical implications. Clin Infect Dis. (1999) 28:1–11. 10028061 10.1086/515079

[10] Kesser BW Hashisaki GT Spindel JH Ruth RA Scheld WM. Time course of hearing loss in an animal model of pneumococcal meningitis. Otolaryngol Neck Surg. (1999) 120:628–37. 10.1053/hn.1999.v120.a92772 10229585

[11] Brandt CT Cayé-Thomasen P Lund SP Worsøe L Østergaard C Frimodt-Møller N. Hearing loss and cochlear damage in experimental pneumococcal meningitis , with special reference to the role of neutrophil granulocytes. Neurobiol Dis. (2006) 23:300–311. 10.1016/j.nbd.2006.03.006 16798006

[12] Klein M Koedel U Pfister H-W Kastenbauer S. Meningitis-associated hearing loss: protection by adjunctive antioxidant therapy. Ann Neurol. (2003) 54:451–8. 10.1002/ana.10684 14520656

[13] Coimbra RS Loquet G Leib SL. Limited efficacy of adjuvant therapy with dexamethasone in preventing hearing loss due to experimental pneumococcal meningitis in the infant rat. Pediatr Res. (2007) 62:291–4. 10.1203/PDR.0b013e318123fb7c 17622952

[14] Grandgirard D Burri M Agyeman P Leib SL. Adjunctive daptomycin attenuates brain damage and hearing loss more efficiently than rifampin in infant rat pneumococcal. Antimicrob Agents Chemother. (2012) 56:4289–95. 10.1128/AAC.00674-12 22644021 PMC3421563

[15] Rivolta MN. New strategies for the restoration of hearing loss: challenges and opportunities. Br Med Bull. (2013) 105:69–84. 10.1093/bmb/lds035 23175701

[16] Dubey A Prajapati KS Swamy M Pachauri V. Heat shock proteins: a therapeutic target worth to consider. Vet world. (2015) 8:46–51. 10.14202/vetworld.2015.46-51 27046995 PMC4777810

[17] Cunningham LL Brandon CS. Heat shock inhibits both aminoglycoside- and cisplatin-induced sensory hair cell death. J Assoc Res Otolaryngol. (2006) 7:299–307. 10.1007/s10162-006-0043-x 16794914 PMC2504613

[18] Martindale JL Holbrook NJ. Cellular response to oxidative stress: Signaling for suicide and survival. J Cell Physiol. (2002) 192:1–15. 10.1002/jcp.10119 12115731

[19] May LA Kramarenko II Brandon CS Voelkel-Johnson C Roy S Truong K. Inner ear supporting cells protect hair cells by secreting HSP70. J Clin Invest. (2013) 123:3577–87. 10.1172/JCI68480 23863716 PMC3967657

[20] Baker TG Roy S Brandon CS Kramarenko IK Francis SP Taleb M. Heat shock protein-mediated protection against cisplatin-induced hair cell death. J Assoc Res Otolaryngol. (2015) 16:67–80. 10.1007/s10162-014-0491-7 25261194 PMC4310861

[21] Ousman SS Tomooka BH van Noort JM Wawrousek EF O'Conner K Hafler DA. Protective and therapeutic role for αB-crystallin in autoimmune demyelination. Nature. (2007) 448:474–9. 10.1038/nature05935 17568699

[22] Van Noort JM Bsibsi M Nacken PJ Verbeek R Venneker EHG. Therapeutic intervention in multiple sclerosis with alpha B-crystallin: A randomized controlled phase IIa trial. PLoS ONE. (2015) 10:1–19. 10.1371/journal.pone.0143366 26599332 PMC4657879

[23] Arrigo A-P Simon S Gibert B Kretz-Remy C Nivon M Czekalla A. Hsp27 (HspB1) and αB-crystallin (HspB5) as therapeutic targets. FEBS Lett. (2007) 581:3665–74. 10.1016/j.febslet.2007.04.033 17467701

[24] Kurnellas MP Brownell SE Su L Malkovskiy AV Rajadas J Dolganov G. Chaperone activity of small heat shock proteins underlies therapeutic efficacy in experimental autoimmune encephalomyelitis. J Biol Chem. (2012) 287:36423–34. 10.1074/jbc.M112.371229 22955287 PMC3476308

[25] Arac A Brownell SE Rothbard JB Chen C Ko RM Pereira MP. Systemic augmentation of αB-crystallin provides therapeutic benefit twelve hours post-stroke onset via immune modulation. Proc Natl Acad Sci USA. (2011) 108:13287–92. 10.1073/pnas.1107368108 21828004 PMC3156222

[26] Hagemann TL Boelens WC Wawrousek EF Messing A. Suppression of GFAP toxicity by αB-crystallin in mouse models of Alexander disease. Hum Mol Genet. (2009) 18:1190–9. 10.1093/hmg/ddp013 19129171 PMC2655774

[27] Bsibsi M Peferoen LAN Holtman IR Nacken PJ Gerritsen WH Witte ME. Demyelination during multiple sclerosis is associated with combined activation of microglia/macrophages by IFN-γ and alpha B-crystallin. Acta Neuropathol. (2014) 128:215–29. 10.1007/s00401-014-1317-8 24997049

[28] Kamradt MC Chen F Cryns VL. The small heat shock protein alpha B-crystallin negatively regulates cytochrome c- and caspase-8-dependent activation of caspase-3 by inhibiting its autoproteolytic maturation. J Biol Chem. (2001) 276:16059–63. 10.1074/jbc.C100107200 11274139

[29] Mehlen P Kretz-Remy C Préville X Arrigo AP. Human hsp27, Drosophila hsp27 and human alphaB-crystallin expression-mediated increase in glutathione is essential for the protective activity of these proteins against TNFalpha-induced cell death. EMBO J. (1996) 15:2695–706. 8654367 PMC450205

[30] Masilamoni JG Jesudason EP Baben B Jebaraj CE Dhandayuthapani S Jayakumar R. Molecular chaperone α-crystallin prevents detrimental effects of neuroinflammation. Biochim Biophys Acta - Mol Basis Dis. (2006) 1762:284–93. 10.1016/j.bbadis.2005.11.007 16443350

[31] Bsibsi M Holtman IR Gerritsen WH Eggen BJL Boddeke E Valk P. Alpha-B-crystallin induces an immune-regulatory and antiviral microglial response in preactive multiple sclerosis lesions. J Neuropathol Exp Neurol. (2013) 72:970–9. 10.1097/NEN.0b013e3182a776bf 24042199

[32] Noort JM Van Bsibsi M Nacken PJ Gerritsen WH Amor S Holtman IR Biomaterials activation of an immune-regulatory macrophage response and inhibition of lung in fl ammation in a mouse model of COPD using heat-shock protein alpha B-crystallin-loaded PLGA microparticles. Biomaterials. (2013) 34:831–40. 10.1016/j.biomaterials.2012.10.028 23117214

[33] Bakthisaran R Tangirala R Rao CM Biochimica et Biophysica Acta Small heat shock proteins : role in cellular functions and pathology. BBA Proteins Proteom. (2015) 1854:291–319. 10.1016/j.bbapap.2014.12.019 25556000

[34] Muri L Grandgirard D Buri M Perny M Leib SL. Combined effect of non-bacteriolytic antibiotic and inhibition of matrix metalloproteinases prevents brain injury and preserves learning, memory and hearing function in experimental paediatric pneumococcal meningitis. J Neuroinflammation. (2018) 15:233. 10.1186/s12974-018-1272-8 30131074 PMC6103863

[35] Leib SL Leppert D Clements J Täuber MG. Matrix metalloproteinases contribute to brain damage in experimental pneumococcal meningitis. Infect Immun. (2000) 68:615–20. 10.1128/IAI.00073-14 10639424 PMC97183

[36] Klopstein A Santos-Nogueira E Francos-Quijorna I Redensek A David S Navarro X Beneficial effects of B-crystallin in spinal cord contusion injury. J Neurosci. (2012) 32, 14478–88. 10.1523/JNEUROSCI.0923-12.2012 23077034 PMC6621445

[37] Liechti FD Grandgirard D Leppert D Leib SL. Matrix metalloproteinase inhibition lowers mortality and brain injury in experimental pneumococcal meningitis. Infect Immun. (2014) 82:1710–8. 10.1128/IAI.00073-14 24491581 PMC3993388

[38] Perny M Solyga M Grandgirard D Roccio M Leib SL Senn P. *Streptococcus pneumoniae* -induced ototoxicity in organ of Corti explant cultures. Hear Res. (2017) 350:100–9. 10.1016/j.heares.2017.04.012 28460251

[39] Kazuo O Grimm CM Corrales CE Senn P Monedero RM Géléoc GSG Differential distribution of stem cells in the auditory and vestibular organs of the inner ear. J Assoc Res Otolaryngol. (2007) 8:18–31. 10.1007/s10162-006-0058-3 17171473 PMC2538418

[40] Masuda S Nakazawa D Shida H Miyoshi A Kusunoki Y Tomaru U. NETosis markers: Quest for specific, objective, and quantitative markers. Clin Chim Acta. (2016) 459:89–93. 10.1016/j.cca.2016.05.029 27259468

[41] Boettcher M Eschenburg G Mietzsch S Jiménez-Alcázar M Klinke M Vincent D. Therapeutic targeting of extracellular DNA improves the outcome of intestinal ischemic reperfusion injury in neonatal rats. Sci Rep. (2017) 7:15377. 10.1038/s41598-017-15807-6 29133856 PMC5684414

[42] Fuchs TA Abed U Goosmann C Hurwitz R Schulze I Wahn V. Novel cell death program leads to neutrophil extracellular traps. J Cell Biol. (2007) 176:231–41. 10.1083/jcb.200606027 17210947 PMC2063942

[43] Masilamoni JG Jesudason EP Bharathi SN Jayakumar R. The protective effect of α-crystallin against acute inflammation in mice. Biochim Biophys Acta - Mol Basis Dis. (2005a) 1740:411–20. 10.1016/j.bbadis.2004.11.002 15949709

[44] Masilamoni JG Vignesh S Kirubagaran R Jesudason EP Jayakumar R. The neuroprotective efficacy of α-crystallin against acute inflammation in mice. Brain Res Bull. (2005b) 67:235–41. 10.1016/j.brainresbull.2005.07.002 16144660

[45] Fujioka M Okano H Ogawa K. Inflammatory and immune responses in the cochlea: potential therapeutic targets for sensorineural hearing loss. Front Pharmacol. (2014) 5:287. 10.3389/fphar.2014.00287 25566079 PMC4274906

[46] Grandgirard D Schürch C Cottagnoud P Leib SL. Prevention of brain injury by the nonbacteriolytic antibiotic daptomycin in experimental pneumococcal meningitis. Antimicrob Agents Chemother. (2007) 51:2173–8. 10.1128/AAC.01014-06 17371820 PMC1891377

[47] Grandgirard D Oberson K Bühlmann A Gäumann R Leib SL. Attenuation of cerebrospinal fluid inflammation by the nonbacteriolytic antibiotic daptomycin versus that by ceftriaxone in experimental pneumococcal meningitis. Antimicrob Agents Chemother. (2010) 54:1323–6. 10.1128/AAC.00812-09 20065062 PMC2825988

[48] Baud V Karin M. Signal transduction by tumor necrosis factor and its relatives. Trends Cell Biol. (2001) 11:372–7. 10.1016/S0962-8924(01)02064-5 11514191

[49] Haake SM Dinh CT Chen S Eshraghi AA Van De Water TR. Dexamethasone protects auditory hair cells against TNFα-initiated apoptosis via activation of PI3K/Akt and NFκB signaling. Hear Res. (2009) 255:22–32. 10.1016/j.heares.2009.05.003 19442713

[50] Wu Q Wang GP Xie J Guo JY Gong SS. Tumor necrosis factor-α-induced ototoxicity in mouse cochlear organotypic culture. PLoS ONE. (2015) 10:e0127703. 10.1371/journal.pone.0127703 26000970 PMC4441368

[51] Aminpour S Tinling SP Brodie HA. Role of tumor necrosis factor-alpha in sensorineural hearing loss after bacterial meningitis. Otol Neurotol. (2005) 26:602–9. 10.1097/00042871-200401001-00548 16015154

[52] Meli DN Coimbra RS Erhart DG Loquet G Bellac CL Täuber MG. Doxycycline reduces mortality and injury to the brain and cochlea in experimental pneumococcal meningitis. Infect Immun. (2006) 74:3890–6. 10.1128/IAI.01949-05 16790761 PMC1489684

[53] Delgado-Rizo V Martínez-Guzmán MA Iñiguez-Gutierrez L García-Orozco A Alvarado-Navarro A Fafutis-Morris M. Neutrophil extracellular traps and its implications in inflammation: an overview. Front Immunol. (2017) 8:81. 10.3389/fimmu.2017.00081 28220120 PMC5292617

[54] Hohlfeld R Kerschensteiner M Meinl E. Dual role of inflammation in CNS disease. Neurology. (2007) 68:S58–S63. 10.1212/01.wnl.0000275234.43506.9b 17548571

[55] Holtman IR Bsibsi M Gerritsen WH Boddeke HWGM Eggen BJL van der Valk P. Identification of highly connected hub genes in the protective response program of human macrophages and microglia activated by alpha B-crystallin. Glia. (2017) 65:460–73. 10.1002/glia.23104 28063173

[56] Kastenbauer S Klein M Koedel U Pfister HW. Reactive nitrogen species contribute to blood-labyrinth barrier disruption in suppurative labyrinthitis complicating experimental pneumococcal meningitis in the rat. Brain Res. (2001) 904:208–17. 10.1016/S0006-8993(01)02164-3 11406118

[57] Tan WJT Thorne PR Vlajkovic SM. Characterisation of cochlear inflammation in mice following acute and chronic noise exposure. Histochem Cell Biol. (2016) 146:219–30. 10.1007/s00418-016-1436-5 27109494

