# Supplementary figures and images for: Anti-inflammatory and Oto-Protective Effect of the Small Heat Shock Protein Alpha B-Crystallin (HspB5) in Experimental Pneumococcal Meningitis
Source: Front Neurol. 2019 Jun 10;10:570. doi: 10.3389/fneur.2019.00570 (PMC6573805; doi:10.3389/fneur.2019.00570)

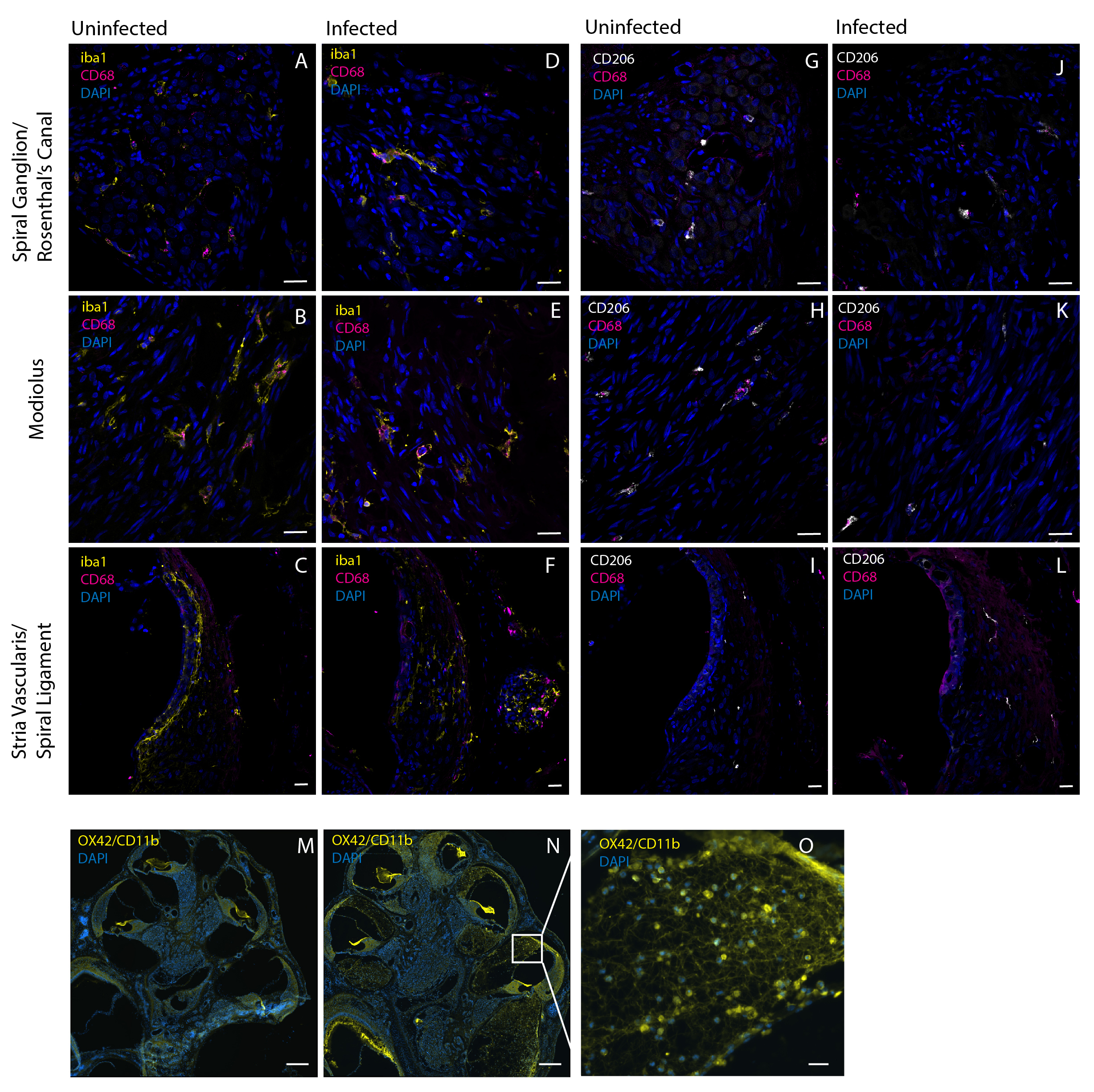

Supplement: Figure S1 — Macrophages in the cochlea. (A–C) Mid-modialar section of uninfected rat cochlea immunostained for CD68 (magenta) and iba1 (yellow), (D–F) Mid-modiloar sections of rat cochlea 42 hours post infection with S. pneumoniae immunostained for CD68 and iba1, (G–I) uninfected cochleae stained for CD68 (magenta) and CD206 (gray), (J–L) cochleae 42 hours post infection stained for CD68 and CD206, scale bars = 20 μm. (M) Representative example of CD11b (yellow) immunostaining and DAPI (blue) in uninfected animals and (N) 42hpi, scale bars = 200 μm (O) detail of the cells in the perilymphatic space, scale bar = 20 μm. [file Image_1.JPEG]
